# Regeneration of Propriospinal Axons in Rat Transected Spinal Cord Injury through a Growth-Promoting Pathway Constructed by Schwann Cells Overexpressing GDNF

**DOI:** 10.3390/cells13131160

**Published:** 2024-07-08

**Authors:** Xiaolong Du, Shengqi Zhang, Aytak Khabbaz, Kristen Lynn Cohen, Yihong Zhang, Samhita Chakraborty, George M. Smith, Hongxing Wang, Amol P. Yadav, Naikui Liu, Lingxiao Deng

**Affiliations:** 1 Department of Neurological Surgery, Indiana University School of Medicine, Indianapolis, IN 46202, USA; dd0341@163.com (X.D.); shengqizhang76@gmail.com (S.Z.); akhabbaz@iu.edu (A.K.); cohenkr@iu.edu (K.L.C.); yizhang@iu.edu (Y.Z.); sachakra@iu.edu (S.C.); 2 Spinal Cord and Brain Injury Research Group, Stark Neurosciences Research Institute, Department of Neurological Surgery, Goodman and Campbell Brain and Spine, Indiana University School of Medicine, Indianapolis, IN 46202, USA; 3 Department of Vascular Surgery, Nanjing Drum Tower Hospital, Affiliated Hospital of Medical School, Nanjing University, Nanjing 210005, China; 4 Department of Rehabilitation Medicine, Zhongda Hospital Southeast University, Nanjing 210009, China; 101012648@seu.edu.cn; 5 Shriners Hospitals Pediatric Research Center, School of Medicine, Temple University, Philadelphia, PA 19140, USA; george.smith@temple.edu; 6 Department of Biomedical Engineering, the University of North Carolina at Chapel Hill, Chapel Hill, NC 27599, USA; apyadav@unc.edu

**Keywords:** descending propriospinal axon, spinal transection, Schwann cell, glial-cell-derived neurotrophic factor, transplantation, regeneration, functional recovery, spinal cord injury, glial response, glial scar

## Abstract

Unsuccessful axonal regeneration in transected spinal cord injury (SCI) is mainly attributed to shortage of growth factors, inhibitory glial scar, and low intrinsic regenerating capacity of severely injured neurons. Previously, we constructed an axonal growth permissive pathway in a thoracic hemisected injury by transplantation of Schwann cells overexpressing glial-cell-derived neurotrophic factor (SCs-GDNF) into the lesion gap as well as the caudal cord and proved that this novel permissive bridge promoted the regeneration of descending propriospinal tract (dPST) axons across and beyond the lesion. In the current study, we subjected rats to complete thoracic (T11) spinal cord transections and examined whether these combinatorial treatments can support dPST axons’ regeneration beyond the transected injury. The results indicated that GDNF significantly improved graft–host interface by promoting integration between SCs and astrocytes, especially the migration of reactive astrocyte into SCs-GDNF territory. The glial response in the caudal graft area has been significantly attenuated. The astrocytes inside the grafted area were morphologically characterized by elongated and slim process and bipolar orientation accompanied by dramatically reduced expression of glial fibrillary acidic protein. Tremendous dPST axons have been found to regenerate across the lesion and back to the caudal spinal cord which were otherwise difficult to see in control groups. The caudal synaptic connections were formed, and regenerated axons were remyelinated. The hindlimb locomotor function has been improved.

## 1. Introduction

After spinal cord injury (SCI), it is difficult to achieve functional recovery. Environmental mechanisms limiting axonal regeneration include (1) a lack of formation of growth-permissive matrices in the lesion site [1]; (2) low regenerative capacity of the mature central nervous system [2,3,4,5]; and (3) impediment to spontaneous axonal growth by the lesion cavity with surrounding glia scar and myelin debris in the extracellular matrix [6,7,8,9]. Combinatory strategy has been applied to target multiple mechanisms [10,11,12,13,14,15]. The most popular strategies, with various degrees of success so far, include providing growth factor or guidance cues in the axonal stump, digesting the glial scar, and stimulating the intrinsic regenerating capacity directly on neuronal soma. Impressive success has been achieved. Different groups of central nervous axons regenerated depending on the variety of treatments [16,17,18,19,20,21,22,23,24,25,26,27].

Schwann cell (SC) transplantation and its combination strategy is one of most promising cell-based strategies for SCI repair [17,21,28,29,30,31,32,33,34,35]. However, critical limitations still exist which seriously retard the application of SC transplantation. One of the main challenges is to promote the growth of regenerated axons through the lesion gap into the host spinal cord so that these axons can make synaptic contacts with target neurons in the distal host spinal cord to reestablish neural circuitry between the damaged spinal cords. This is key to enable the signals from the brain to extend not only into the injured spinal cord but also caudal to the repair site, thus enabling the signals to find to their appropriate targets.

One of the reasons that axons find it difficult to cross the implant/spinal cord interfaces is the reaction of astrocytes and their production of some inhibitory molecules which usually surround the grafted Schwann cells and impede their migration [36,37]. The other reason is that graft environment is more favorable to the regenerated axons than the surrounding host environment which also prevents the regenerated axons from further growth [37,38]. Different efforts have been made to either break the glia barrier in the graft–host interface [14,20,21,23,39,40] or provide the extra growth-promoting signal in the host environment to lure the axonal growth back to the host cord [41,42,43,44,45,46]. Previously, we found that glial-cell-derived neurotrophic factor (GDNF) can not only directly promote axonal regeneration but also affect the interaction between grafted Schwann cells and host reactive astrocytes so that astrocytes form less proteoglycan and become less inhibitory and more permissive to axon growth [47]. Therefore, with GDNF, we can target different barriers which retard axonal regeneration. In the model of partial SCI, we further constructed an axonal growth-promoting pathway by grafting SC-GDNF from the lesion cavity extending to the caudal spinal cord. Although descending propriospinal tract axons (dPST axons) are able to grow beyond the implant and make connections (synapses) with host nerve cells, as partial functional recovery has been achieved [17], this approach may face a much more difficult scenario in complete spinal cord transection injury, as other strategies do [10,48,49,50,51,52]. The model of spinal cord transection injury is not usually chosen in spinal cord injury research due to the extreme difficulty of overcoming the large lesion gap caused by significant retraction of broken stumps, unstable graft–host interface, strong glial scar, and severe neuronal damage. Spared axons are not available to contribute to the plasticity and recovery of function that can occur after partial injuries [53,54,55,56]. However, it is the gold standard to prove the efficacy of a strategy toward true axonal regeneration across the lesion gap [57]. Furthermore, the majority of human injuries are severe in extent (“ASIA A”), which corresponds to complete and permanent loss of function below the lesion [58,59]. Therefore, the model of complete transection spinal cord injury is highly clinically relevant. It is necessary to test any promising strategy in this most severe model. We therefore transfer the application of successful SCs-GDNF transplantation strategy from a spinal cord hemisection model to a complete spinal cord transection model in adult rats.

## 2. Method and Materials

### 2.1. Generation of Purified Schwann Cells (SCs)

SCs were purified as described previously [17]. The epineurium was carefully removed with fine forceps and the nerve was cut into 1 mm-long explants. The explants were cultured in Dulbecco’s Modified Eagle’s Medium (DMEM) with 10% fetal bovine serum (FBS; Life Technologies, Grand Island, NY, USA) for one week to allow fibroblast migration to occur. The explants were transferred to new dishes weekly for six weeks until all fibroblasts had migrated out of the explant and pure SCs were observed migrating from the nerve segment. Next, the nerve explants were transferred to a culture dish containing 1.25 U/mL dispase (Boehringer Mannheim Biochemicals, Indianapolis, IN, USA) and 0.05% collagenase (Worthington Biochemicals Corp., Freehold, NJ, USA) and incubated overnight at 37 °C in 5% CO_2_. Then, the explants were dissociated, and the isolated cells were seeded onto poly-L-lysine-coated T75 flasks and cultured in DMEM/10% FBS. After two days, the culture medium was replaced with DMEM/10% FBS plus 20 μg/mL bovine pituitary extract (BTI, Stoughton, MA, USA) and 2 μM forskolin (Sigma, St Louis, MO, USA) to enhance SC replication. Once the cells reached approximately 80% confluency, they were washed with DMEM and treated briefly with 0.05% trypsin (Life Technologies, USA) and 0.02% EDTA (Life Technologies, USA). DMEM/10% FBS was added to stop the reaction and the cells were centrifuged, resuspended in fresh DMEM/10% FBS with pituitary extract and forskolin, and plated at a density of ~2 × 106 cells/flask. SC purity (determined as the % S100-positive cells) was quantified. Purified SCs (purity > 98%) at the third or fourth passage were collected for in vitro infection and transplantation.

### 2.2. Transduction of SC In Vitro 

SCs were pre-treated with 4–6 µg/mL polybrene (Sigma-Aldrich, St. Louis, MO, USA) for 30–60 min, infected by lentiviruses expressing either green fluorescence protein (SCs-GFP) or GDNF (SCs-GDNF) at multiplicity of infection (MOI) of 4, resulting in about 50% infection of cells [47,60].

### 2.3. Animal Groups and Exclusion Criteria 

An initial set of 51 adult female Sprague Dawley (SD) rats (180–200 g, Harlan, Indianapolis, IN, USA) were randomly divided into three groups (n = 17). To ensure that the spinal cords were completely transected, the following highly conservative exclusive criteria were used [17]: (1) incomplete interruption of GFAP-labeled astrocytes in the spinal cord examined in a set of sections spaced 100 µm apart; (2) a close distance between the BDA tracer injection site and the lesion (<3 mm); (3) the animals did not survive all three major surgeries (spinal transection, cell transplantation, and tracer injection), and all their behavior scores were excluded from analysis. Using these criteria, five rats in the group that received caudal injection of DMEM rats were excluded (three died after surgery and two were observed to have BDA leaking, final n = 12). Three rats in the group that received caudal injection of SCs-GFP were excluded (two died after surgery and in one we observed BDA leaking, final n = 14). One rat in the group that received caudal injection of SCs-GDNF was excluded (died after surgery, final n = 16). An additional subgroup of six rats received the same injury but received the graft of SCs, which received the double infection of two different lentiviruses to express GFP and GDNF (SCs/GFP-GDNF) into the lesion cavity and SCs-GDNF into the caudal host cord. The purpose of this subgroup is to display the survival of grafted SCs and their interaction with the host astrocytes. Another subgroup of six rats received the same injury but with graft of SCs expressing GFP (SCs-GFP) into the lesion cavity as a control for the SCs/GFP-GDNF group to determine whether SCs, combined with or without GDNF, had any effect on the behavior of host reactive astrocytes such as migration within the lesion gap. To keep all researchers blinded to the treatment groups during behavioral assessments and surgeries, we established a standard practice of coding all animals with numbers that were randomized and not reflective of treatment groups. Information about the type of treatment was separated from the coded numbers immediately following treatment and was not present during these procedures [17].

Experimental protocols were approved by the Institutional Animal Care and Use Committee (IACUC) of the Indiana University School of Medicine in accordance with the NIH Guidelines for the Care and Use of Laboratory Animals. All efforts were made to minimize the number of animals used and their suffering.

### 2.4. Thoracic Spinal Cord Transection Injury and Cell Transplantation

**Figure 1:**
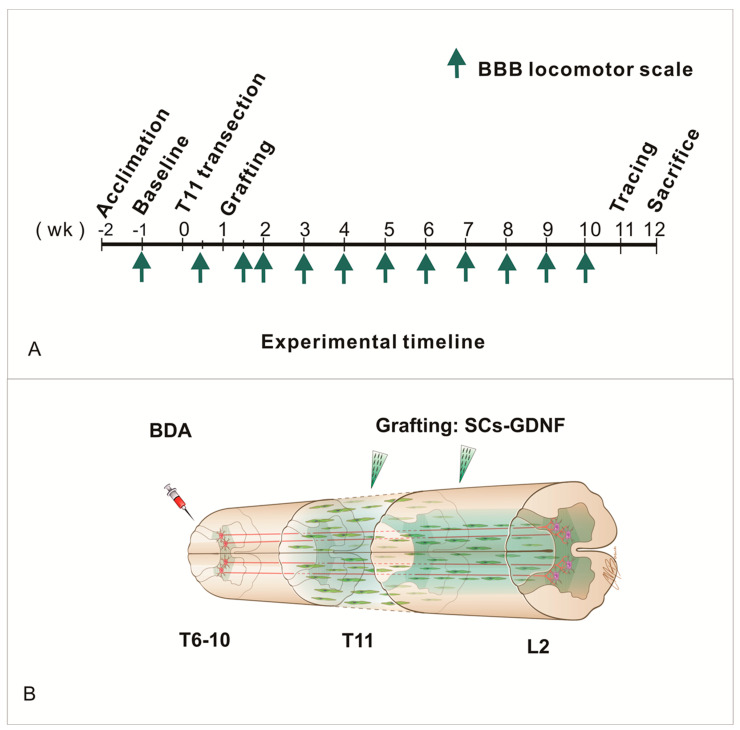
(**A**) Timeline of experimental design. (**B**) Schematic description of a continuous axonal growth-promoting pathway. Transplantation of Schwann cells overexpressing glial-cell-line-derived neurotrophic factor inside both the lesion and caudal host cord forming an axonal growth pathway extending from the axonal cut ends to the site of innervation in the distal spinal cord.

Female Sprague Dawley (SD) rats were anesthetized with an intraperitoneal injection of a ketamine (40–95 mg/kg)/xylazine (5–10 mg/kg) cocktail. All surgical procedures were performed under sterile conditions and all animal care was provided in accordance with the guidelines provided by the Institutional Animal Care and Use Committee (IACUC) of the Indiana University School of Medicine in accordance with the NIH Guidelines for the Care and Use of Laboratory Animals. Lacrilube ophthalmic ointment (Allergan Pharmaceuticals, Irvine, CA) was applied to the eyes, and the antibiotic Bicillin (C-R, 20,000 U, A. J. Buck, Inc., Owings Mills, MD, USA), triple antibiotic ointment Bacitracin (400 units/gram), Neomycin 3.5 mg/gram, and Polymyxin B Sulfate (5000 units/g) were administered to the surgical wound. The procedures for spinal cord transection and cell transplantation were described in previous publications [17]. After a multilevel laminectomy was performed to expose T10–12 cord segments, the dura was incised longitudinally, and the spinal cord was transected at T11. After complete transection, rostral and caudal spinal stumps were automatically retracted to leave a gap of 2–3 mm. The completeness of injury was judged by seeing the interior surface of the vertebrate bodies. The dura and muscle were sutured. One week after injury, the spinal cord was re-exposed. A combination of 6 μL fibrinogen solution (25 mg/mL) with SCs-GDNF (1 × 10^5^ cell/μ) was injected into the lesion cavity in all groups, and then 6 μL thrombin solution (25 U/mL) was injected into the same site to solidify the cellular bridge [61]. For different groups, DMEM, SCs-GFP, or SCs-GDNF was bilaterally injected into the caudal host spinal cord correspondingly. The injections were made at 0.5 and 1.0 mm caudal to the edge of lesion and at 0.5 mm lateral to the midline and 1.0 mm ventral to the dorsal surface of the spinal cord. At each injection point, 1.0 µL of DMEM or SCs (-GFP or -GDNF; density: 1.0 × 10^5^ cell/µL) in DMEM was injected (Figure 1B).

### 2.5. Behavior Assessments

The Basso, Beattie, and Bresnahan (BBB) locomotor rating scale was performed one week before the surgery and every week after SCI for up to nine weeks after transplantation (ten weeks after injury).

### 2.6. Anterograde Tracing 

On the 10th week after Schwann cell transplantation, bilateral and stereotaxic injections of biotinylated-dextran amine (BDA; 10%, 1 μL/site; ThermoFisher, Waltham, MA, USA) were made into the intermediate gray matter of the T9–T10 cord at distances of 3–6 mm rostral to the graft (for BDA, 1 injection/site/mm, total 3 injections/side) according to a previously published work [17].

### 2.7. Immunohistochemistry 

Polyclonal rabbit anti-glial fibrillary acidic protein (GFAP) antibody (1:100, Millipore Bioscience Research Re-agents, Burlington, MA, USA), monoclonal mouse anti-synaptophysin antibody (1:1000; Millipore Bioscience Research Reagents, Burlington, MA, USA), rabbit monoclonal to Myelin Protein Zero (P0) antibody (1:500, abcam, Cambridge, MA, USA) were used to identify astrocytes, presynaptic components, regenerated serotonin, and tyrosine hydroxylase axons, respectively, according to an existing protocol [17].

### 2.8. Electron Microscopy 

Briefly, rats were perfused with 2% paraformaldehyde and 1% glutaraldehyde followed by 5% sucrose in phosphate buffer. Sections underwent a freeze–thaw procedure, incubated in avidinbiotin-peroxidase complex (1:50; Vector Laboratories, Newark, CA, USA) containing 2% normal goat serum in PBS, visualized with nickel-DAB/H_2_O_2_, and followed by conventional processes for transmission electron microscopy [17].

### 2.9. Quantification of BDA-Labeled Axons

For quantification of BDA-labeled axons, one set of serial BDA-stained sagittal sections cut at a thickness of 20 μm spaced at 100 μm between adjacent sections’ cable were used for analysis. We defined the host–graft boundary by GFAP staining. We set the starting point “0” located inside the lesion which has an equal distance to the rostral and caudal boundary. The number of BDA-labeled axons on each section was counted and summed in lesion epicenter “0” point, caudal graft–host interface, every 400 μm interval to the caudal interface in the host caudal spinal cord. Percentage of axons which grow across the caudal graft–host boundary was calculated by the equation: Percentage = the number of axons at a specific different distance to the caudal graft–host interface ÷ the number of axons appearing in the “0” point × 100%.

### 2.10. Assessments of GFAP Immunoreactivity In Vivo

Fluorescence intensity of GFAP immunoreactivity (IR) was measured to estimate the fold change in GFAP at the lesion border across the groups. The GFAP-IR was used as a marker to outline the boundary between the grafted and host tissues. After outlining of the astrogliotic region at the graft–host interfaces, the intensity of GFAP-IR in the territory 1 mm caudal to interface was measured by using an Olympix digital camera and NIH Image J 1.53e. For each animal, 20 µm serial sections at equal medio-lateral distances were used for analysis. Sections from each group were processed with the same camera exposure time for fluorescence signal. The total intensity values were then averaged for each group.

### 2.11. Statistical Analysis

Data are presented as mean ± SD. Two-way with Tukey’s post hoc analysis was used to determine statistical significance for BBB evaluation of axonal regeneration. One-way ANOVA was used to determine the significance for glial response. All statistical values were calculated using GraphPad Prism 5.0 software (GraphPad), with a *p* value < 0.05 considered statistically significant.

## 3. Results

Descending propriospinal axons contributed to axonal regeneration through and beyond the lesion.

**Figure 2:**
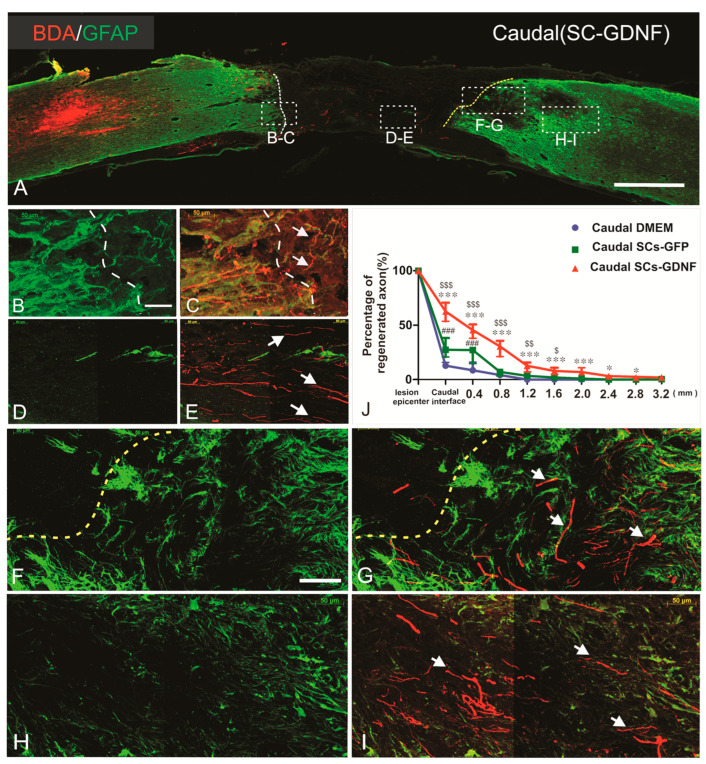
Transected descending propriospinal tract (dPST) axons regenerate into and beyond T11 transection lesion site after treatment with SCs-GDNF graft in the lesion site and caudal beyond the lesion. (**A**) A montage image for BDA-labeled dPST axons (red), host astrocytes (GFAP, green) in a sagittal section at low magnification of a case receiving SCs-GDNF transplantation into the lesion gap at T11 and injection of the SCs-GDNF into the distal host spinal cord. BDA injection site was located 3 mm rostral to the lesion. Regions at different sites were boxed and examined at higher magnification at rostral graft–host interface (**B**,**C**), within the graft (**D**,**E**), caudal graft–host interface (**F**,**G**), and cord spinal cord proper (**H**,**I**) in the subsequent images. (**B**,**C**) At the rostral host–graft interface, regenerated dPST axons (white arrows in (**C**,**E**,**G**,**I**)) crossed the host–graft boundary (white dash line) and grew into the graft. (**D**,**E**) Within the SCs-GDNF graft, continued growth of regenerated dPST axons was found (white arrows in (**E**)). (**F**,**G**) At the distal graft–host interface (yellow dash line in (**F**,**G**)), regenerated axons (white arrows in (**G**)) penetrated into the caudal host cord. (**H**,**I**) Within the distal host spinal cord, regenerated dPST axons (white arrows in (**E**)) were clearly seen. (**J**) Percentage of regenerated axons appearing in the caudal host cord over the regenerated axon appearing in the graft epicenter at different distances from caudal graft–host boundary among three groups. *** *p* < 0.001, * *p* < 0.05, the distal SCs-GDNF group versus the distal DMEM group; ### *p* < 0.001, distal SCs-GFP group versus distal DMEM group. $$$ *p* < 0.001, $$ *p* < 0.01, $ *p* < 0.05, distal SCs-GDNF group versus distal SCs-GFP group. Scale bars: (**A**), 1000 µm; (**B**–**I**), 50 µm. Animal number: caudal injection of DMEM group n = 12; caudal injection of SCs-GFP group n = 14; caudal injection of SCs-GDNF group n = 16. Bar heights represent means ± SD.

Previously, in a spinal cord hemisection model, we found that most of the dPST axons regenerating across the lesion originated from the neurons whose soma was located in lamina VII and VIII of the T8–10 spinal cord levels. To trace the course of regenerated propriospinal axons directly through and beyond the graft in a complete transection model, we injected an anterograde tracer biotinylated-dextran amine (BDA, 2%) into the intermediate gray matter of the T9–T10 spinal cord at distances 3–6 mm rostral to the graft. The axons originating from T9 propriospinal neurons were labeled by BDA. According to strict criteria that we established, two rats in the DMEM group and one rat in the SCs-GFP group were excluded due to BDA leaking into graft. Strict criteria have been used to judge the true regeneration in our study [17,18,57]. After spinal cord complete transection, a large lesion gap was creased with GFAP immunoreactivity (IR) clearly seen at the edge of the rostral and caudal host stumps (Figure 2A). The regenerated axons exhibit irregular trajectories with abrupt turns (Figure 2G,I) rather than existing in tightly linear bundles typical of spared axons. Histological evidence indicated that all rats had complete transection injuries. Most of the regenerated axons appearing in the lesion gap and caudal host cord were distributed within the territory of grafted SCs-GDNF (Figure 2). The regenerating axons back to the host spinal cord were preferred to the gray matter that lacked adult CNS myelin, a major molecular inhibitor of axonal growth. Regenerated axons within the graft and caudal host cord displayed unpredicted orientation rather than a rostral–caudal longitudinal pattern commonly seen in normal or sham-operated spinal cord. In control groups in which either SCs or DMEM were injected into the distal host tissue, outgrowth was sparse and limited to within 800 µm of the graft–host interface characterized by GFAP staining. In the DMEM group, only 12.43 ± 2.3% of total regenerated axons (544.6 ± 41.94) inside the graft epicenter passed across the caudal interface (n = 12). In the SC group, 27.82 ± 6.81% of total regenerated axons inside the graft epicenter (583.6 ± 61.42) passed across the caudal interface (n = 14). In contrast, in the SCs-GDNF group, a significantly greater number (61.40 ± 6.15%) of total regenerated axons inside the graft epicenter (611.43 ± 54.42) penetrated though the distal graft–host interface (n = 16). The longest dPST axon that extended into the caudal cord was found to be at 3.2 mm from the distal interface (Figure 2J, Supplemental Figure S1). Without GDNF treatment, most of the regenerated dPST axons stopped in the caudal graft–host interface, although these axons can regenerate into the grafted SCs environment (Supplemental Figure S2).

### 3.1. Regenerated Axons Formed Synapse on Caudal Host Spinal Cord

**Figure 3:**
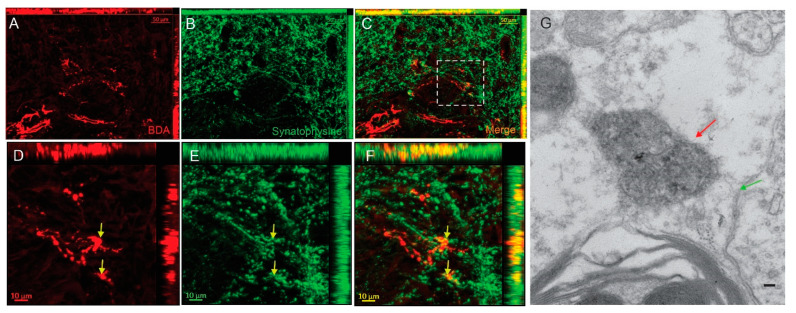
Regenerated dPST axons formed synapse on host neurons in the distal host spinal cord. (**A**–**C**) BDA-labeled dPST axons (red) colocalize with the presynaptic marker synaptophysin (SYN) (green). (**D**,**E**,**F**) Higher-magnification images from dash square area in (**C**) showed the colocalization (yellow arrows) between bouton structure (red) and synaptophysin-stained structure (green). (**G**) Immuno-electron microscopy showed that a regenerated dPST presynaptic terminal, containing dark reaction product of BDA (red arrow) formed a synapse with a host dendritic spine (green arrow). Scale bars: (**A**–**C**) 50 µm; (**D**–**F**) 10 µm; (**G**) 500 nm.

To determine whether regenerated axons form new synapses on target neurons in distal host spinal cord, immunostaining for presynaptic marker synaptophysin was performed. Colocalization of BDA-labeled axons with synaptophysin was observed (Figure 3A–F). To provide more solid evidence for synaptic formation, we performed immuno-electron microscopy to identify BDA immunoreactivity at the EM level. We found that BDA-labeled regenerated axons formed presynaptic terminals, which were electron-dense after immunolabeling for BDA reacted with streptavidin-horseradish. These presynaptic terminals made contacts with postsynaptic components of other neurons/terminals (Figure 3G).

### 3.2. Remyelination Was Enhanced by SC Overexpressing GDNF

**Figure 4:**
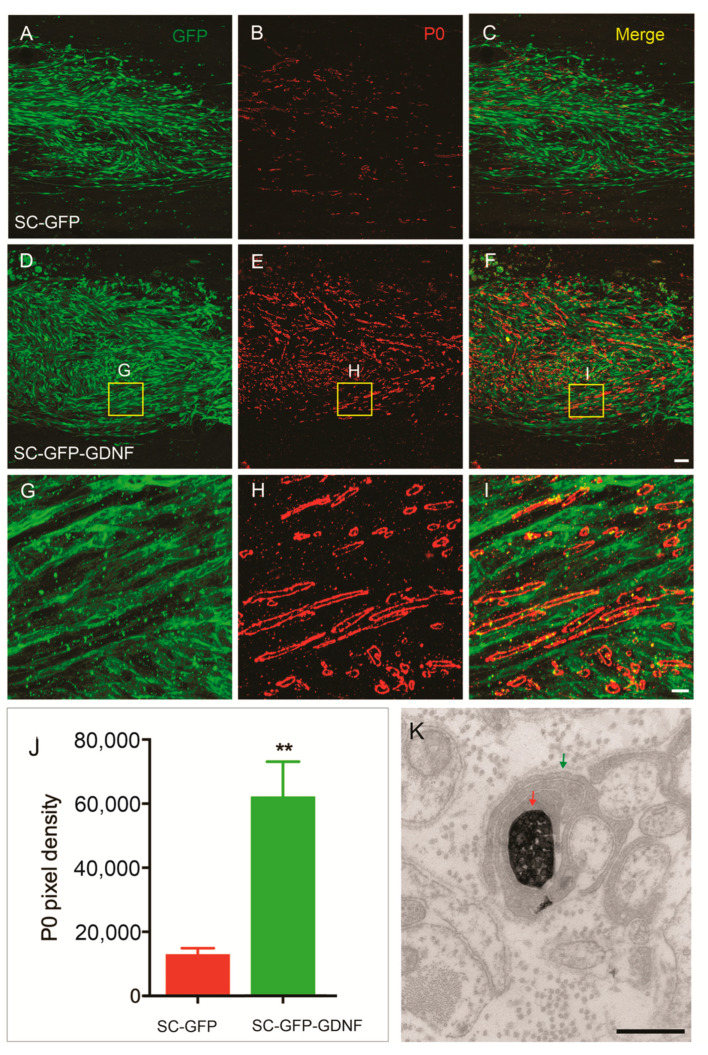
SCs overexpressing GDNF promoted the myelination within the transplant. (**A**–**C**) P0-positive myelination in SC expressing GFP (SC-GFP) in control group. (**D**–**F**) Many more myelinations found in SC expressing GDNF graft. (**G**–**I**) Higher-magnification images from (**D**–**F**) showed the relationship of GFP SCs with P0 positive myelin. (**J**) Quantitative analysis showed P0 intensity was increased in the graft of SC-GDNF compared with in the graft of SC-GFP, ** *p* < 0.01; (**K**), Immuno-electron microscopy confirms that regenerating BDA-containing dPST axons (red arrow) were being myelinated (green arrow). Scale bar: (**A**–**F**) = 50 μm; (**G**–**I**) = 5 μm. (**K**) = 500 nm.

We evaluated the ability and extent of SCs to myelinate axons that grew into the graft by quantifying the fluorescence intensity of myelin protein-zero (P0)-positive immunolabeling within the SC graft (Figure 4). SC overexpressing GDNF significantly enhanced the remyelination of regenerating axons (62,141 ± 10,868), compared with the SC-only group (13,055 ± 1877) (*p* < 0.01).

### 3.3. SCs-GDNF Remodeled Astrocytic Responses at the Caudal Graft–Host Interfaces

**Figure 5:**
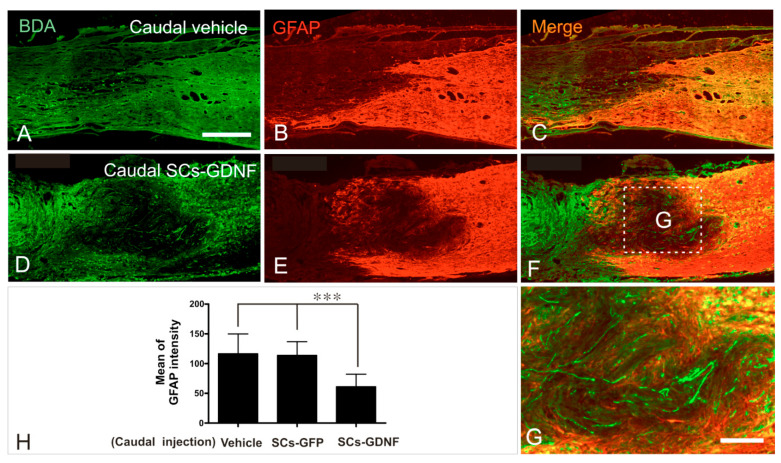
SCs-GDNF grafted within and caudal to a spinal transection modified astroglial responses at the caudal graft–host interface. Representative photomicrographs of GFAP expression in the caudal graft–host interface in a case that received DMEM (**A**–**C**) or a case that received SCs-GDNF (**D**–**F**) injections into the caudal host spinal cord. In the DMEM-injected case, increased expression of GFAP (**B**,**C**) was found at the distal graft–host interface. In contrast, in the SCs-GDNF-injected case, the expression of GFAP was considerably reduced (**E**,**F**). Such reduction was correlated with significant regeneration of dPST axons into the caudal host spinal cord (**D**,**F**). High magnification of boxed area in (**F**) was further appreciated in (**G**) colocalization of regenerated axons (BDA, red). (**H**) Quantitative analyses show that the difference in GFAP expressions in the three groups was statistically significant (***: *p* < 0.001). Scale bar: (**A**–**F**) 500 μm; (**G**) 150 μm.

**Figure 6:**
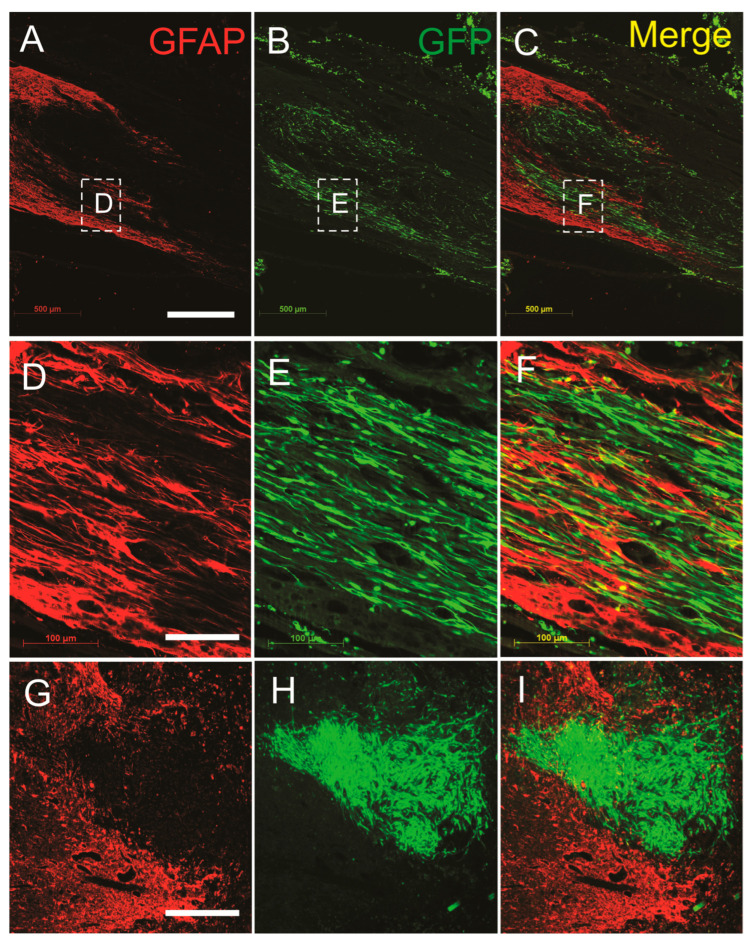
GDNF, expressed by SCs-GFP, promoted bidirectional migration of astrocytes into the graft and SCs into the host. (**A**–**C**) Low magnification shows the bidirectional migration of astrocytes and SCs towards each other at the rostral graft–host interface. (**D**–**F**) High magnification of boxed area in (**A**–**C**) shows the intermingling of astrocyte and SCs, creating a blurring boundary. (**G**–**I**) In the absence of GDNF, grafted GFP-expressing Schwann cells (SCs-GFP, green) and host astrocytes (GFAP-IR, red) were separated by a sharp boundary. Scale bar: (**A**–**C**) 500 μm; (**D**–**I**) 50 μm.

GFAP is a hallmark of reactive astrogliosis. In our previous hemisection model, we have found that GDNF significantly reduced glial response along the grafted SCs/host astrocytes interface [47]. We confirmed this phenomenon here in the transection model. The graft of SCs-GDNF significantly inhibited the astrocytic glial response in caudal host spinal cord, evidenced by obviously dim immunofluorescence staining of GFAP (Figure 5). This attenuation of astrocytic glial response may facilitate the growth of regenerated axons back to the caudal host spinal cord (Figure 5). Contrarily, in the control group with the caudal graft of vehicle or SCs expressing GFP, the glial response did not decrease (Figure 5, Supplemental Figure S3). Quantification data show the significant difference of caudal GFAP intensity among the three groups (Figure 5; vehicle vs. SCs-GDNF *p* < 0.001; SCs-GFP vs. SCs-GDNF *p *< 0.001). Interestingly, the astrocytes in the area grafted with SCs-GDNF (inside the lesion and caudal cord) were characterized by a relatively smaller cellular soma and slim, long cellular processes and loose cellular distribution (Figure 5 and Figure 6), significantly different from the astrocytes in the area without SCs-GDNF graft which displayed hypertrophic cellular morphology and dense cellular organization (Figure 5).

### 3.4. SCs-GDNF Promoted Migration of Host Astrocytes into the SCs-GDNF-Filled Lesion Gap Where They Are Closely Associated with Regenerated dPST Axons

**Figure 7:**
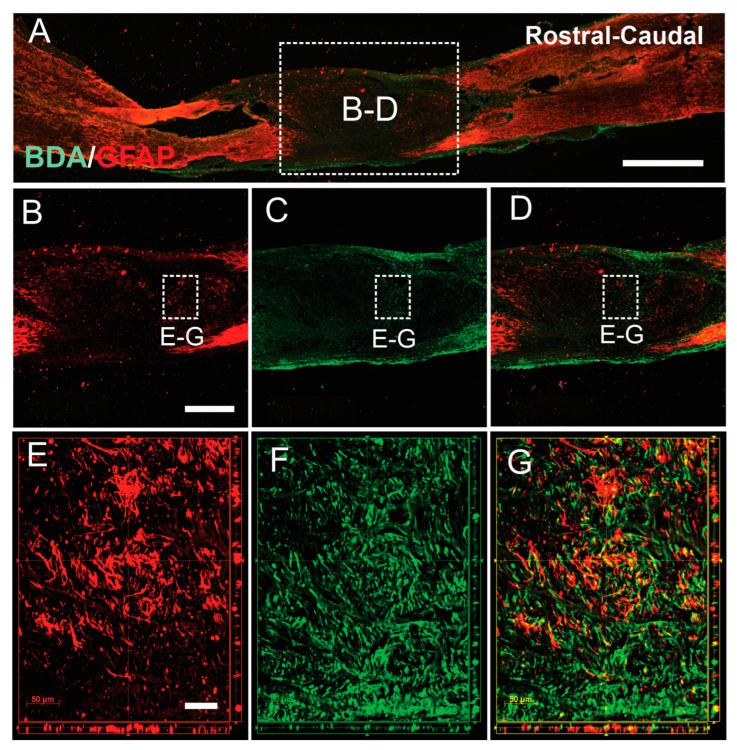
GDNF-induced parallel alignment between migratory astrocytes and regenerated axons. (**A**–**D**) Low magnification images showed migration of host astrocytes (GFAP IR red) into the SCs-GDNF graft. (**E**–**G**) At high magnification, a significant amount of regenerated descending propriospinal tract (dPST) axons (**F**, BDA-labeled, green) were shown in close association with the host astrocytes migrated into the graft region (**E**, GFAP-IR, red) in the XY, XZ, and YZ planes. Scale bar: (**A**) 1000 μm; (**B**–**D**) 100 μm; (**E**–**G**) 50 μm.

We then evaluated the effects of SCs-GDNF on the migratory ability of astrocyte, stained with GFAP, into the SCs-GDNF-filled lesion gap from the host side of the spinal cord. In all the groups that received SCs overexpressing GDNF, robust migration of astrocytes into the transplants was observed (Figure 6). These migrations caused blurring of the graft–host interface. In the subgroup of the transplantation of SCs-GFP into the lesion gap, a dense meshwork of reactive astrocytes (GFAP-IR) was found surrounding the graft SCs (Figure 6). The migrated astrocytes were characterized by elongated processes and rostral caudal orientation inside the graft (Figure 6). Migratory astrocytes were closely associated with regenerated BDA-labeled descending propriospinal axons (Figure 7), while in our subgroups to determine the survival of grafted SCs in the lesion gap or caudal to it, survival of GFP-positive SCs within the lesion gap was clearly seen and they were surrounded by a dense meshwork of reactive astrocytes (GFAP-IR), indicating that transplantation of SCs alone, without GDNF, may trigger astrocytic responses that sharply separate these two populations of glial cells at the lesion site (Figure 6).

### 3.5. The Constructed SCs-GDNF Growth-Promoting Pathway Promoted Improved Hindlimb Locomotor Function 

**Figure 8:**
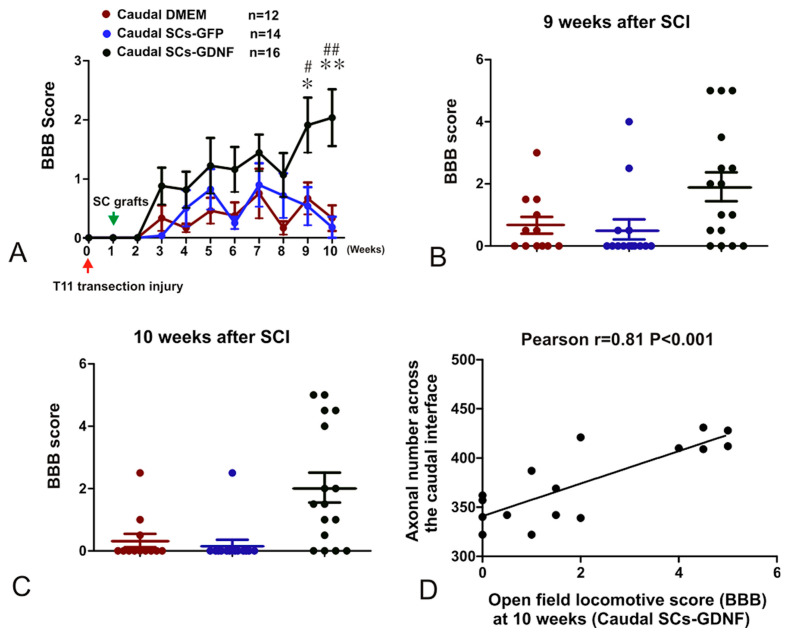
Partial recovery of hindlimb motor function after axonal regeneration through and beyond a continuous SCs-GDNF growth-promoting pathway established after a complete spinal transection. (**A**) Improved BBB locomotor recovery was found in the group that received intraspinal transplantation and caudal injection of SCs-GDNF (green), compared with caudal injection of either SCs-GFP (blue) or DMEM (red) (** *p* < 0.01, * *p* < 0.05, SCs-GDNF vs. DMEM; ## *p* < 0.01, # *p* < 0.05, SCs-GDNF vs. SCs-GFP). (**B**,**C**) Scatter plot showing BBB score of different groups in 9th week (**B**) and 10th week (**C**) after spinal transection. (**D**) Correlation between number of regenerating axons across the caudal boundary and the open field locomotive score at 10th week. Bar heights represent means ± SD.

To determine the functional efficacy of the SCs-GDNF transplantation within and caudal to the graft, the Basso, Bettie, and Bresnahan (BBB) locomotor rating scale was applied every week after injury (Figure 1). No significant difference between the three groups that received caudal injection of the SC-GDNF, SC-GFP, or vehicle was found at or before eight weeks post-injury (Figure 8A). At nine weeks post-SCI, the average score in the vehicle control group (n = 12) was 0.67 ± 0.93 (mean ± SD). In the group that received caudal injections of SC-GFP, the average score was 0.54 ± 1.2 (n = 14). In the group that received caudal injection of SC-GDNF, the average score was 1.91 ± 1.86 (n = 16). At 10 weeks post-SCI, the average score in the vehicle control group (n = 12) was 0.33 ± 0.75 (mean ± SD). In the group that received caudal injections of SC-GFP, the average score was 0.17 ± 0.67 (n = 14). In the group that received caudal injection of SC-GDNF, the average score was 2.03 ± 1.92 (n = 16) (Figure 8B,C). The average BBB score of the SC-GDNF group was significantly higher than that of the other two groups (*p* < 0.001). The group with SC-GFP transplants did not exhibit any significant improvements in the BBB score when compared with the vehicle control group. The results did not indicate that the recovery reached the plateau at nine weeks after the transplantation, the time point at which testing was discontinued. There was correlation between individual functional recovery and number of regenerated BDA-labeled descending propriospinal axons which grew across the caudal graft–host interface (r = 0.81, *p* < 0.001) (Figure 8).

## 4. Discussion

A continuous growth-promoting pathway of SCs-GDNF promoted regeneration of specific descending propriospinal axons through and beyond a spinal cord transection. At the level of the thoracic spinal cord, without any exogenous growth factors, only a small number of sensory and propriospinal axons regenerated into either the SCs or PN graft environment in transection injury [28,62]. To promote regeneration of long descending central nerve axons, a stronger signal should be conveyed from the broken axonal stump to the neuronal soma to awaken the regenerative capacity [63].

Neurotrophic factors are strong candidates for combination therapies, given their known ability to promote neuronal survival, axonal regeneration/sprouting and neuroprotection, and SC differentiation, and to enable growth of neurites on inhibitory substrates [64,65,66]. The response of different neuronal tracts to neurotrophic factors depends on the repertoire of receptors expressed as well as the signals elicited by these factors [67]. GDNF protein promoted sensory and propriospinal axons’ regeneration in the model of partial injury [17,68]. In the current work, long-term caudal distribution of a high concentration of GDNF provided stimulating signals in the caudal inhibitory milieu, which consequently significantly attracted propriospinal axons to regenerate into the distal host spinal cord. There exists the possibility that other supraspinal descending pathways may also contribute to the overall regeneration stimulated by the SCs-GDNF growth-promoting pathway.

### 4.1. GDNF Modulate the Interaction between Grafted SCs and Host Astrocytes

A significant challenge of SCs transplantation strategy is the poor integration between grafted SCs and host astrocytes. Astrocytes and SCs normally reside separately in the PNS and CNS, respectively. At the peripheral nerve entry zone, astrocytes contribute to the formation of glial limitans that prevents SCs from migrating into the CNS and generates barriers to axonal regeneration after injury [69,70]. This segregation also occurred after transplantation of SCs into the astroglial environment in the spinal cord [71,72]. Exogenous SCs can aggravate the astroglial response induced by the injury. The strong glial production, such as proteoglycans built up along the graft–host interface, at least partially accounted for the segregation between grafted SCs and host astrocytes [73]. This glial scar not only limits the mutual migration between SCs and astrocytes but also retards the axonal regeneration into the SCs environment [74]. Furthermore, the poor integration of SCs to the surrounding host tissue has trapped regenerated axons within the graft, causing presumably non-functional regeneration [10]. Interestingly, we previously found GDNF can modify the glial environment in a spinal cord hemisection and bridge transplantation of the SCs-GDNF model [47]. Our current work confirmed that with GDNF treatment, the abrupt boundary often seen in SCs graft has been replaced by zigzagged or an even unidentified interface. The large number of migratory astrocytes was observed dispersed amongst and in close association with grafted SCs in the territory full of GDNF within both the lesion and caudal graft site, which indicated that SCs and astrocytes are less repellent to each other under the influence of GDNF. Such irregular borders are more permissive for axons to grow from the host astrocyte environment to the grafted SC territory as well as back to the host cord [19,20,31,47,68,75].

### 4.2. Astrocytes within GDNF Territory Display a Permissive Character for Axonal Regeneration

There are many clues that the permissibility for axonal growth of astrocytes is related with differentiated status, which is usually reflected by their morphological pattern [76,77]. Radial glia, the immature cell that supports axonal growth during development, has an elongated, bipolar phenotype, whereas mature astrocyte exhibits multiple processes and primarily expresses GFAP [78]. In vitro, the glial-precursor-derived astrocytes cultured with bone-morphogenetic protein also displayed extended processes which promote axon regeneration. The astrocytes appearing in the developmental phase can create several adhesive molecules permissive for axonal regeneration [77]. After CNS injury, reactive astrocytes become hypertrophied and enhance expression of GFAP and other glial components inhibitory to axon regeneration while subpopulation of reactive astrocyte expresses nestin, an embryonic marker indicating an immature status although in a temporary and minor manner [79]. Several growth factors and cytokines can significantly keep astrocytes in the morphology characterized by slim and thinner processes, reduced GFAP expression, and marked transformation to a bipolar morphology similar to a relatively undifferentiated state permissive to axonal growth [31,73,76,80]. Early studies of complete transection with SCs transplantation reported axons sprouting short distances into the lesion in association with sporadic migratory astrocytes. These axonal sprouts were enclosed within a basal lamina continuous from these migratory astrocytes and grafted SCs. These tunnel-like structures are distinct from the inhibitory sheets of basal lamina found in CNS scar tissue [31]. These permissive post-traumatic astrocytes may be derived from de-differentiating and/or de novo astrocytes. Our current results indicate that under GDNF in situ treatment, the significantly attenuated glial response was found and accompanied by transformed astrocyte morphology, especially those migratory astrocytes with bipolar distribution of slim processes and reduced GFAP expression both in the lesion gap and caudal area of transplantation. These migratory astrocytes associated with transplanted SCs escorted regenerating axons across the lesion. Thus, agreeing with the de-differentiation phenomenon discussed in previous studies, we found that GDNF switches the astrocyte from inhibitory phenotype with a stellate morphology to a growth-permissive phenotype with an elongated or radial-like morphology.

### 4.3. Limitation of the Study

There are some limitations in this study. Traditionally, we harvested the SCs from female rats and transplanted into the female SCI model. Considering that the majority of SCI patients are male, it is necessary to evaluate the gender effect on SC behavior in this transplantation strategy. In addition, increased locomotor functional recovery did not reach a plateau. Considering the long-term survival of SC observed in our previous study, it would be necessary to observe the promotional effect of SC transplantation over a longer period. Lastly, supraspinal axonal regeneration, which is very important for functional recovery, was not significant. Alternative strategies, such as combination with other neurotrophic factors, degradation of glial scar, or direct stimulation of supraspinal neurons, should be considered in future study.

## 5. Conclusions

Following a spinal cord complete transection and construction of a SCs-GDNF growth-promoting pathway through and beyond a lesion gap of the thoracic cord, we observed that descending dPST axons regenerated through and beyond the lesion gap. In the distal host spinal cord, regenerated axons formed synapses and were myelinated. Such structural regeneration occurred in close association with an enhanced but limited locomotor recovery. With the GDNF treatment, regenerating axons appeared closely associated with substrates expressing both inhibitory and stimulatory molecules, which suggested that axonal regeneration can take place in situations where both signals are present. For a therapeutic intervention to work, it needs to shift the balance between these two opposing signals. As the major component in the glial scar, astrocytes are heterogeneous cells. Some populations display axonal growth-inhibitory characteristics, but some are permissive. The high astrocytic responses to local cues make them promising candidates as therapeutic targets to shift to a dominant beneficial pattern. GDNF combined with SCs transplantation can not only directly support axonal regeneration but also transform reactive astrocyte to be permissive. These dual effects endorse GDNF as having very powerful potential in repairing spinal cord injury.

## Data Availability

Data will be available from the corresponding author upon reasonable request.

## Supplementary Materials

The following supporting information can be downloaded at: https://www.mdpi.com/article/10.3390/cells13131160/s1. Figure S1: The number of regenerated dPST axons that regenerated into the caudal host cord at different distances from the caudal graft-host interface. ***, *p* < 0.001, *, *p* < 0.05, SC-GDNF (red) versus DMEM (blue); ###, *p* < 0.001; #, *p* < 0.05, SC-GFP (green) versus DMEM (blue). Bar heights represent means ±SD. Figure S2:Descending propriospinal tract (dPST) axons regenerated into the SCs-GDNF graft but stopped at the caudal graft-host interface in the absence of SCs-GDNF in the distal host spinal cord. (A) A montage image of a sagittally-cut spinal cord showing the lesion site stained with BDA for regenerated dPST axons (red), and GFAP for astrocytes (green). (B-D) High magnification of the boxed area in A at the caudal graft-host interface shows that regenerated dPST axons (white arrows, B and D) failed to growth into the distal host spinal cord defined by a reactive astrocyte border (yellow dashed line, B-D). Figure S3: Schwann cells expressing GFP (SCs-GFP) injected into the distal host spinal cord survived amongst host astrocytes. (A) presence of SCs-GFP in the distal host spinal cord (green). (B) Host astrocytes (GFAP-IR, red), (C) Merge image indicated that SCs-GFP had no effect on the astrocytic expression of GFAP. Scale bar: 50 μm.
