# Supplementary figures and images for: Regeneration of Propriospinal Axons in Rat Transected Spinal Cord Injury through a Growth-Promoting Pathway Constructed by Schwann Cells Overexpressing GDNF
Source: Cells. 2024 Jul 8;13(13):1160. doi: 10.3390/cells13131160 (PMC11240522; doi:10.3390/cells13131160)

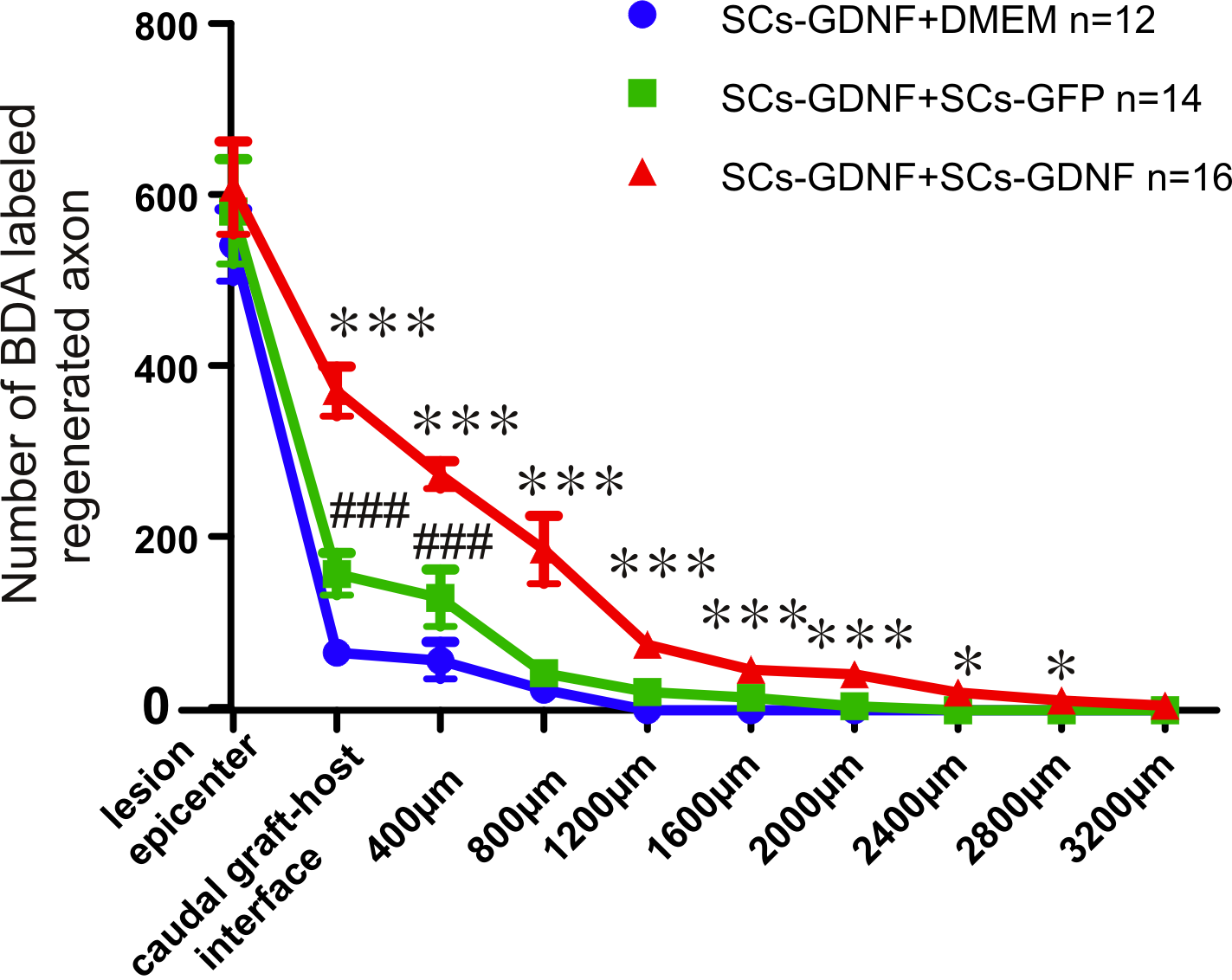

Supplement: Supplementary file 1 [file cells-13-01160-s001.zip › Supple. 1.tif]

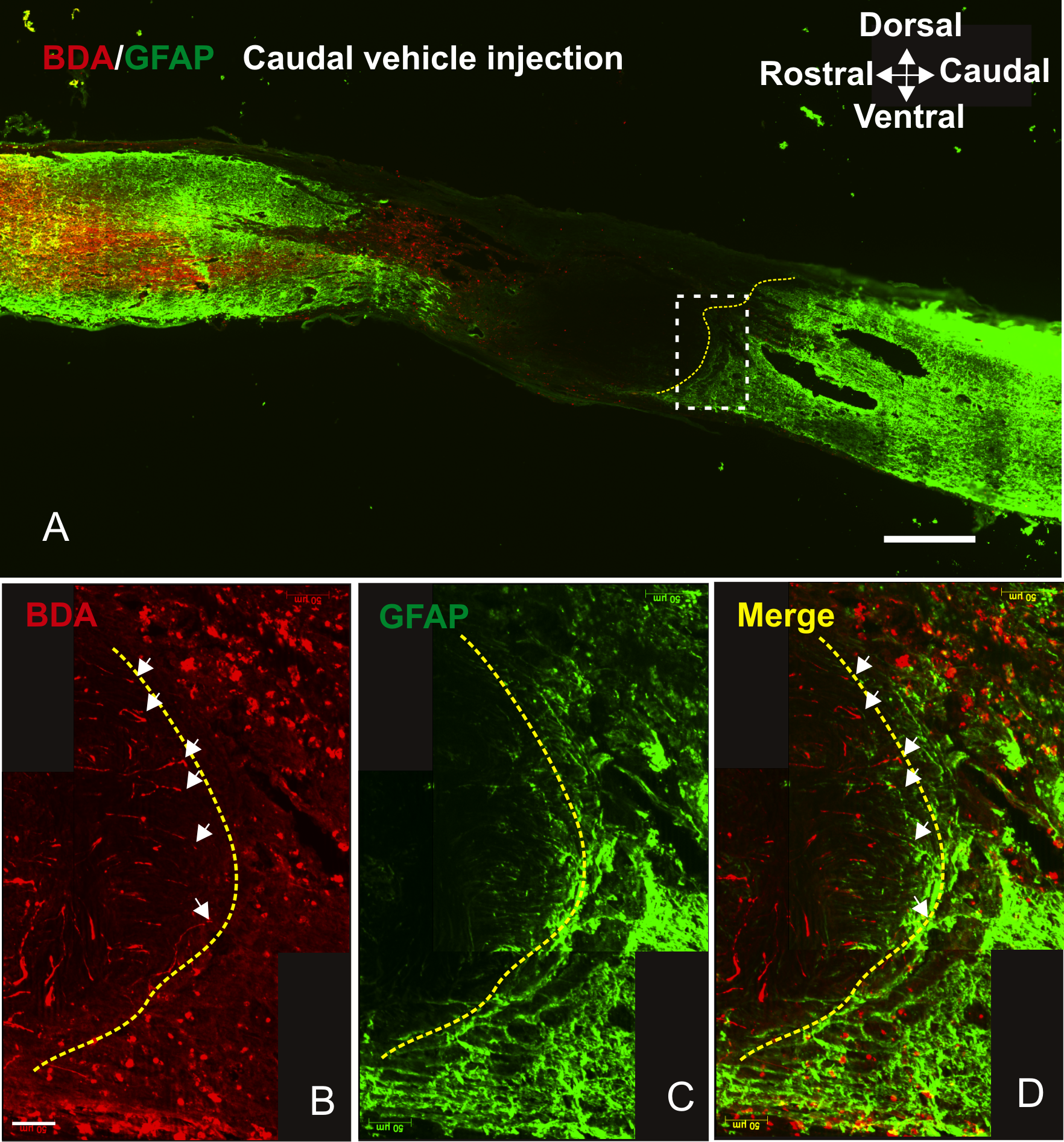

Supplement: Supplementary file 1 [file cells-13-01160-s001.zip › Supple. 2.tif]

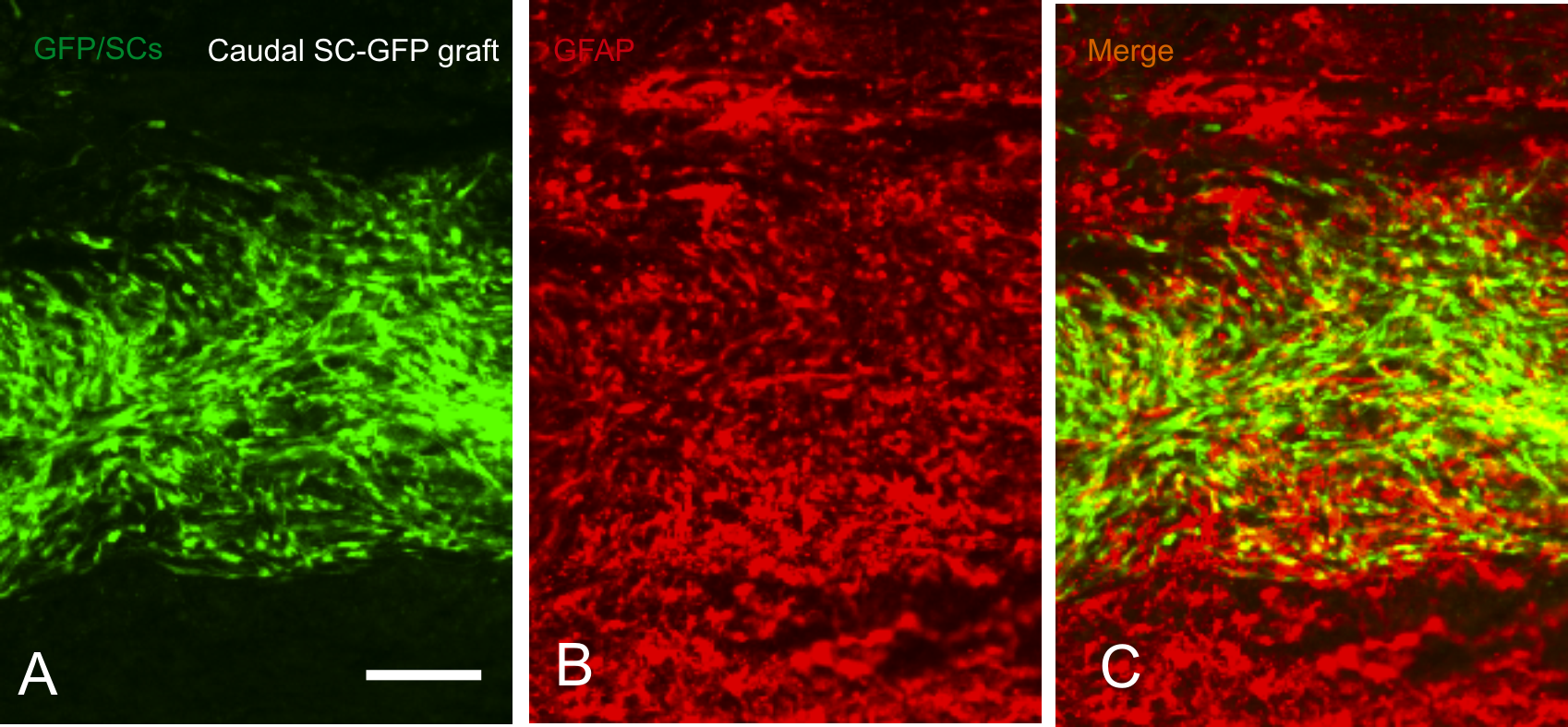

Supplement: Supplementary file 1 [file cells-13-01160-s001.zip › Supple. 3.tif]
